# Fornix deep brain stimulation induced long-term spatial memory independent of hippocampal neurogenesis

**DOI:** 10.1007/s00429-016-1188-y

**Published:** 2016-02-01

**Authors:** Sarah Hescham, Yasin Temel, Sandra Schipper, Mélanie Lagiere, Lisa-Maria Schönfeld, Arjan Blokland, Ali Jahanshahi

**Affiliations:** 1 Department of Neuroscience, Maastricht University, P.O. Box 616, 6200 MD Maastricht, The Netherlands; 2 Department of Neurosurgery, Maastricht University, P.O. Box 616, 6200 MD Maastricht, The Netherlands; 3 Department of Neurology, Maastricht University, P.O. Box 616, 6200 MD Maastricht, The Netherlands; 4 Department of Neuropsychology and Psychopharmacology, Maastricht University, P.O. Box 616, 6200 MD Maastricht, The Netherlands; 5 Department of Morphology, Biomedical Research Institute (BIOMED), Hasselt University, Hasselt, Belgium; 6 European Graduate School of Neuroscience (Euron), Maastricht, The Netherlands

**Keywords:** Deep brain stimulation, Fornix, Memory, Hippocampus, Neurogenesis

## Abstract

Deep brain stimulation (DBS) is an established symptomatic treatment modality for movement disorders and constitutes an emerging therapeutic approach for the treatment of memory impairment. In line with this, fornix DBS has shown to ameliorate cognitive decline associated with dementia. Nonetheless, mechanisms mediating clinical effects in demented patients or patients with other neurological disorders are largely unknown. There is evidence that DBS is able to modulate neurophysiological activity in targeted brain regions. We therefore hypothesized that DBS might be able to influence cognitive function via activity-dependent regulation of hippocampal neurogenesis. Using stimulation parameters, which were validated to restore memory loss in a previous behavioral study, we here assessed long-term effects of fornix DBS. To do so, we injected the thymidine analog, 5-bromo-2′-deoxyuridine (BrdU), after DBS and perfused the animals 6.5 weeks later. A week prior to perfusion, memory performance was assessed in the water maze. We found that acute stimulation of the fornix improved spatial memory performance in the water maze when the probe trial was performed 1 h after the last training session. However, no evidence for stimulation-induced neurogenesis was found in fornix DBS rats when compared to sham. Our results suggest that fornix DBS improves memory functions independent of hippocampal neurogenesis, possibly through other mechanisms such as synaptic plasticity and acute neurotransmitter release.

## Introduction

Deep brain stimulation (DBS) by permanently implanted electrodes in the brain is a popular treatment. Up to now, DBS has provided treatment for more than 130,000 patients worldwide, suffering from Parkinson’s disease, essential tremor, and dystonia (Benabid et al. 1991; Blond and Siegfried 1991; Holloway et al. 2006). Despite its widespread use, the precise mechanism of action of DBS therapy remains unknown. Clinical applications of DBS in neuropsychiatric diseases including memory impairment are in the early stages or under investigation (Temel et al. 2012; Holtzheimer and Mayberg 2011). The fornix has gained growing attention as potential DBS target to alleviate memory impairments (Hamani et al. 2008; Fontaine et al. 2013; Laxton et al. 2010). In a phase 1 clinical trial, the effects of chronic forniceal stimulation in Alzheimer’s disease (AD) patients were assessed (Laxton et al. 2010). Although some patients showed improvement and slowing in the rate of cognitive decline at 6 and 12 months, others did not respond to the therapy. It was reported recently that in two AD patients with the best clinical response in this study, bilateral fornix DBS of 130 Hz, 3–3.5 V and 90 µs pulse width increased hippocampal volume (Sankar et al. 2014). This hippocampal volume change strongly correlated with hippocampal metabolism and a volume change in the fornix and mammillary bodies, suggesting a circuit-wide effect of stimulation.

Besides inducing morphological changes, fornix DBS might also have an effect on adult neurogenesis. Throughout life, new neurons are continuously generated in the subventricular zone and in the granule cell layer of the dentate gyrus. Most of these adult-generated dentate granule cells are thought to contribute to the formation of hippocampus-dependent memory (Shors 2008; Deng et al. 2010). A few studies in rodents already provided evidence that DBS of the anterior thalamic nucleus and entorhinal cortex promotes hippocampal neurogenesis by increasing the cell proliferation and survival of newly generated neurons in the dentate gyrus (Encinas et al. 2011; Stone et al. 2011; Toda et al. 2008). In these studies, rats or mice were first treated with electrical stimulation in the target region, then injected with the cell proliferation marker 5-bromo-2′-deoxyuridine (BrdU) and sacrificed after a few weeks. Increased neurogenesis could furthermore be linked to improved spatial memory in the water maze (Stone et al. 2011; Tronel et al. 2015).

In the present study, we investigated whether fornix DBS induces long-term neurogenic changes in the dentate gyrus by injecting BrdU after DBS. Subsequent to the completion of a hippocampus-dependent memory task, we analyzed the hippocampi 6 weeks after the first injection of BrdU and labeled them with antibodies against BrdU and the neuronal marker, neuron-specific nuclear protein (NeuN).

## Materials and methods

### Subjects

**Fig. 1:**
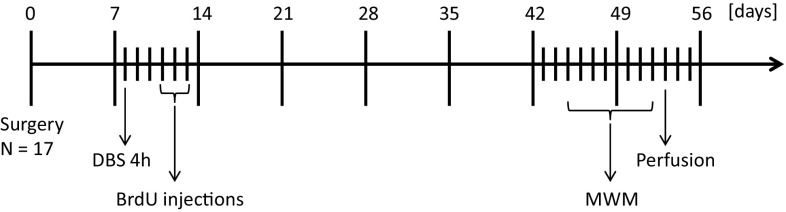
Timeline of the experimental procedures expressed in days. One week following surgery, fornix DBS rats (*n* = 10) received stimulation for 4 h, while sham rats (*n* = 7) were only attached to cables and not stimulated. BrdU injections were given 3 days later twice per day for a period of 3 days. Morris water maze (MWM) testing included training, probe trials and reversal training and all rats were sacrificed the following day

Sprague–Dawley rats from Charles River (Sulzfeld, Germany) were used, their weight ranging between 280 and 300 g at the time of surgery. The temperature of the colony room was maintained at a temperature of 21 ± 1 °C. Rats were housed two or three per cage with rat chow and water available ad libitum in a reversed 12:12 h light–dark cycle. For the time of experimental procedures (stimulation and BrdU injections) rats were housed individually, but were re-grouped thereafter. All animal procedures were executed during the dark phase. Experiments were approved and carried out in accordance with the Animal Experiments and Ethics Committee of Maastricht University. An exact timeline of the procedures can be found in Fig. 1.

### Experimental groups

Rats were randomly assigned to one of the following experimental groups: sham (*n* = 7) or fornix DBS (*n* = 10). We included more rats in the fornix DBS group to account for incorrect electrode placements.

### Surgical procedure

The exact surgical procedure as well as details for DBS electrodes have been described elsewhere (Tan et al. 2010). In short, DBS electrodes were implanted in the fornix using a rodent stereotactic apparatus (Stoelting, Wood Dale, IL, USA, model 51653). The rat skull was exposed under isoflurane anesthesia (IsoFlo^®^, Abbott Laboratories Ltd, Berkshire, Great Britain) and two burr holes for bilateral DBS electrodes were made at the level of the fornix (coordinates from bregma according to the rat brain atlas of Paxinos and Watson (Paxinos and Watson 2006): AP: −1.8 mm; ML: 1.3 mm; DV: −8.0 mm). The construct was permanently anchored with dental cement (Paladur, Heraeus Kulzer GmbH, Hanau, Germany) until animals were sacrificed. Sham rats underwent the same electrode implantations, but were not stimulated.

### Deep brain stimulation

DBS was applied for 4 h at 100 Hz, 100 µA and 100 µs pulse width using a digital stimulator (DS8000, WPI, Berlin, Germany) and separate stimulus isolators for each of the bilateral electrodes (DLS100, WPI, Berlin, Germany). Sham animals were only attached to cables and not stimulated.

### BrdU labeling

BrdU was used to identify cells that started to proliferate after DBS treatment. BrdU (Sigma-Aldrich) was dissolved in 0.9 % NaCl (pH 7.6) to 8 mg/ml. Three days after DBS, all rats were injected intraperitoneally twice daily (8 h apart) with 50 mg/kg BrdU for 3 consecutive days. The interval between DBS and onset of BrdU injection was chosen based on a previous study, in which proliferative activity evaluated by BrdU in the dentate gyrus reached a plateau at 3–5 days after DBS (Stone et al. 2011).

### Water maze

The water maze consists of a circular black polyethylene tank (diameter 153 cm) with 63 cm-high walls. The pool was filled with 40 cm of water, which was maintained at 22 ± 1 °C and made opaque by adding black, nontoxic paint. The black escape platform (diameter 11 cm) was submerged 1.5 cm below the surface of the water. A video camera was mounted in the center above the pool and registered movements of the rat (Ethovision Pro, Noldus, The Netherlands).

All animals received four trials during four acquisition sessions, which were given on consecutive days (total of 16 swim trials). Different start locations were used and tracing began when the animal was released into the pool facing the wall of the tank. Rats were given 60 s to reach the platform and if the rat failed to find the platform within 60 s, the experimenter guided the rat to the target. The time between subsequent trials was about 10 min. A probe trial was given 1 h and 48 h after the last trial on the fifth acquisition session.

In the reversal learning paradigm, the platform was moved to a novel location and the rats were again trained for two consecutive days with four trials/day. Rats were subjected to the reversal probe trial, 1 h after the last training session.

### Tissue collection

At the end of the experiments, the rats were overdosed with pentobarbital (Apotheek Faculteit Diergeneeskunde, Utrecht, The Netherlands). Transcardial perfusions with Tyrode buffer and then Somogyi fixative solution (4 % paraformaldehyde, picric acid, PBS, glutaraldehyde) were carried out. Brains were removed and placed in fresh fixative (identical composition as Somogyi solution, but lacking glutaraldehyde) at 4 °C for 2 h. Subsequently, brains were transferred to 1 % NaN_3_ at 4 °C for long-term storage.

For vibratome sectioning (Leica^®^, Wetzlar, Germany), brains were embedded in 10 % gelatin from porcine skin (Sigma-Aldrich, Zwijndrecht, The Netherlands) and then cut into 30 µm slices in the frontal plane. Slices were immediately transferred into 1 % NaN_3_ and kept at 4 °C.

### Immunohistochemistry

Based on our previous studies (Jahanshahi et al. 2013), a selection of six or seven animals per group is sufficient to make statistical comparisons in stereological analyses of immunohistochemical readouts. Therefore, we have performed a double-immunofluorescent BrdU/NeuN staining for sections containing the hippocampal formation in seven randomly selected fornix DBS and seven sham animals. For BrdU detection, DNA denaturation was conducted by incubating for 2 h in 50 % formamide at 65 °C, followed by washing and 30 min in 2 N HCl at 37 °C. After blocking with donkey serum, sections were incubated with mouse monoclonal anti-BrdU (1:100; Sigma-Aldrich, Zwijndrecht, Netherlands) overnight at 4 °C. Subsequently, donkey anti-mouse secondary antibody (1:100; Alexa 488, Invitrogen, Carlsbad, CA) was applied. Incubation with biotinylated NeuN (1:100; Chemicon, Temecula, CA) was carried out for 3 days at 4 °C, followed by streptavidin 594 (1:1000 Invitrogen, Carlsbad, CA). Lastly, brain sections were mounted and coverslipped with 80 % glycerol.

The number of BrdU/NeuN double-labeled cells was counted using the stereological procedure, optical fractionator. Counts were done using a confocal microscope (DSU, Olympus^®^ BX51W1), a motorized stage, and the StereoInvestigator software (MicroBrightField, Williston, VT). The granule cell layer of the dentate gyrus was defined as the region of interest. All double-labeled BrdU/NeuN cells in an average of six sections, 300 µm apart, were counted with a 60× objective. The chosen brain sections extended from bregma −3.1 mm to bregma −4.9 mm. The total number of positive cells was estimated as a function of the number of cells counted and the sampling probability (Schmitz and Hof 2000).

### Verification of electrode placements

Sections containing electrode trajectories from all animals were mounted on gelatin-coated glass slides. A standard hematoxylin–eosin staining was employed to inspect the sections using bright field microscopy.

### Statistical analyses

Statistical analysis was performed using SPSS (SPSS Inc., Chicago, IL, USA). Acquisition data of water maze trials were analyzed by repeated measures ANOVA. Probe trials and immunohistochemical findings were analyzed using an independent samples *t* test. All *p* values <0.05 were considered to be statistically significant.

## Results

### Verification of electrode placements

**Fig. 2:**
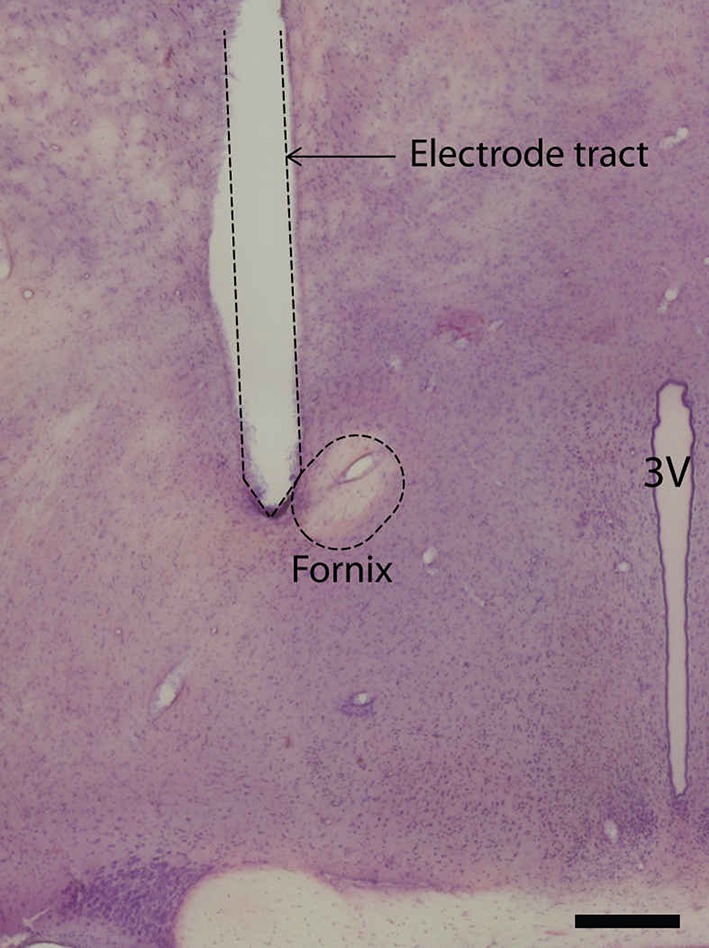
Illustrative coronal section showing the histological verification of the electrode location in the vicinity of the fornix (*scale bar* 500 μm). 3V, third ventricle

DBS electrodes were all correctly placed in the vicinity of the fornix (Fig. 2). The electrode trajectory in one animal of the stimulated group was located ventrally to the fornix, but since the rat showed similar behavior to other stimulated animals, we expected that the fornix was within the electric field emitted by the electrode and included the rat in the analysis.

### Water maze

**Fig. 3:**
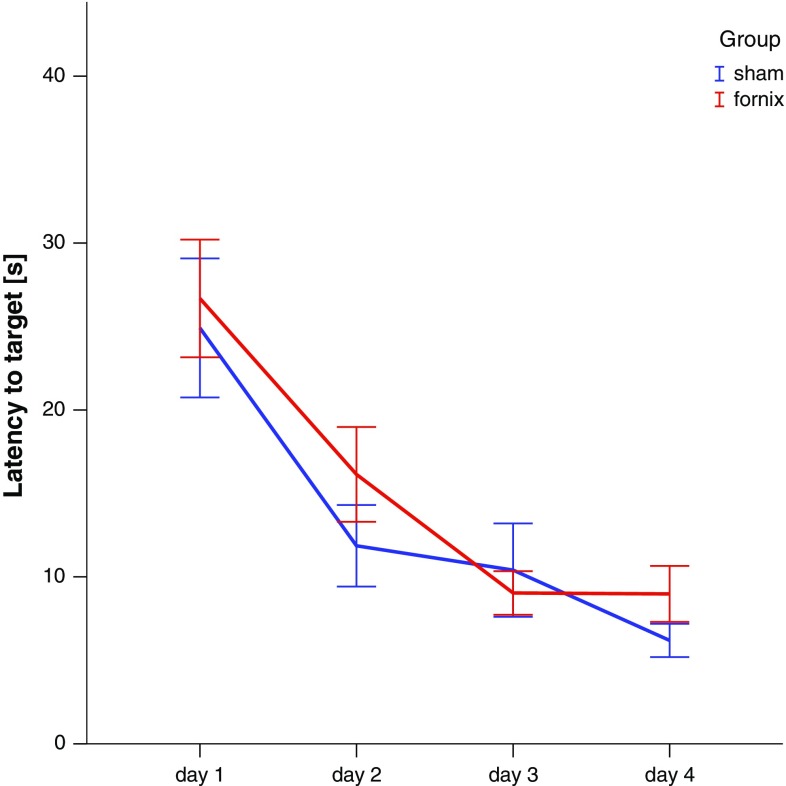
Acquisition trials of fornix DBS and sham animals. No difference in the latency to find the platform was found between the groups. Data represent mean ± S.E.M

**Fig. 4:**
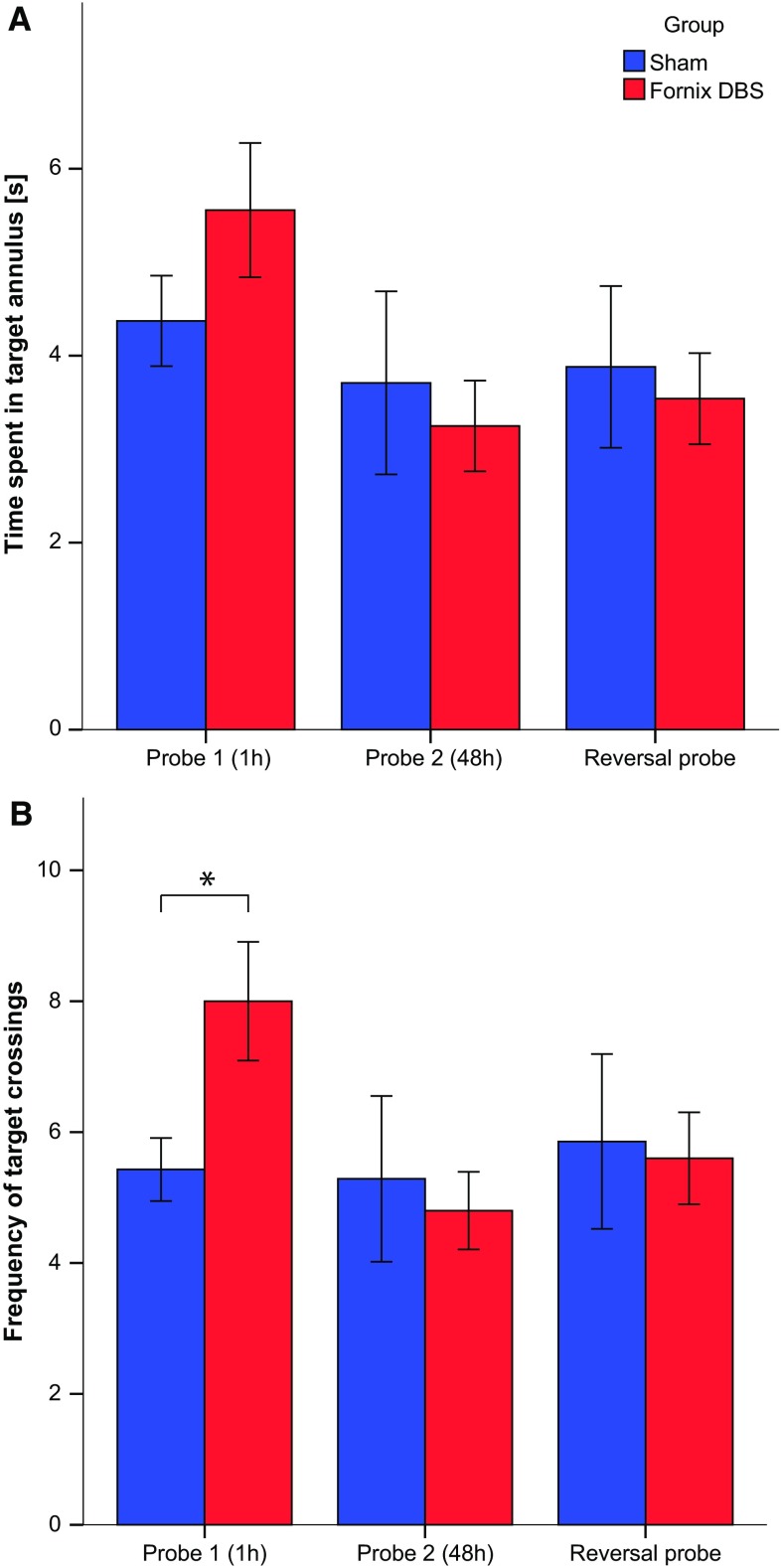
Effects of fornix DBS during the different probe trials (after 1 or 48 h delay and in the probe trial 1 h after the reversal training) when compared to sham. There was no difference between the groups in time spent in the target annulus (**a**). Fornix DBS rats, however, crossed the target annulus more often than sham animals (**b**). *Asterisk* indicates *p* < 0.05. Data represent mean ± S.E.M

During the acquisition phase, latency to find the platform declined similarly in both groups [*F*(1,66) = 0.658; n.s., Fig. 3]. One hour after completion of the training, spatial memory was assessed in a probe trial. In this test, both DBS and sham rats spent similar time in the target zone [*t*(15) = 1.241; n.s, Fig. 4a]. However, fornix DBS rats crossed the former platform location more frequently when compared to sham [*t*(15) = 2.209; *p* < 0.05, Fig. 4b], indicating that fornix stimulation facilitated short-term spatial memory. At the 48 h probe test, no difference between time spent and number of crossings to the target location was found. In the reversal training, both groups performed equally well and there was also no statistical difference between the groups in the reversal probe trial which took place 1 h after the last reversal training session [all *t*’s ≤ 0.462; n.s.].

### Confocal stereological cell counting

**Fig. 5:**
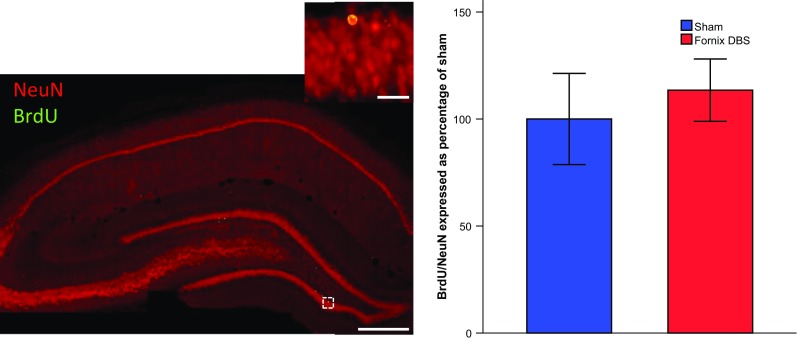
Representative picture (*scale bar* 500 µm) of a hippocampal section stained for NeuN (*red*) and BrdU (*green*) with a high-power inset at the top right showing a double-labeled cell (*scale bar* 50 µm). Graph represents number of double-labeled BrdU/NeuN cells in the dentate gyrus expressed as percentage of sham ± S.E.M. There was no significant difference between the groups

Similar numbers of BrdU/NeuN double-labeled cells in both fornix DBS and sham groups [*t*(12) = 0.522, n.s.] were counted, without any additional differences between the anterior, medial and posterior portions of the dentate gyrus [all *t*’s ≤ 0.985; n.s., Fig. 5].

## Discussion

Adult hippocampal neurogenesis has long been implicated as important for learning and memory (Deng et al. 2010). Consequently, the role of neurogenesis in the adult brain might open up important opportunities for the development of therapeutic interventions, which could moderate or delay disorders with memory decline. Studies in rodents have demonstrated that DBS of the anterior thalamic nucleus or entorhinal cortex increases cell proliferation in the dentate gyrus (Stone et al. 2011; Toda et al. 2008). These targets, however, have not yet been explored as potential treatment for dementia. DBS of the fornix, on the other hand, has already been reported in a case study (Fontaine et al. 2013) and phase I clinical trial (Laxton et al. 2010) to slow down progression of memory loss in individuals with early Alzheimer’s disease. The exact mechanisms of action are yet to be defined. In a previous study, we have also found superior memory performance of fornix DBS rats in an experimental model of dementia (Hescham et al. 2013). We were able to link restoration of memory loss to an increased neuronal activation in the CA1 and CA3 subfield of the dorsal hippocampus, as well as to increased hippocampal acetylcholine levels (Hescham et al. 2015). The hippocampus receives abundant cholinergic innervation from the basal forebrain and it has been shown that selective neurotoxic lesion of the basal forebrain reduces neurogenesis in the dentate gyrus and thus impairs spatial memory (Mohapel et al. 2005). In the same study, systemic administration of the cholinergic agonist physostigmine was able to increase neurogenesis in the dentate gyrus. In contrast to this study, we did not find any evidence for enhanced neurogenesis following fornix DBS.

It has been shown that hippocampal acetylcholine primarily influences proliferation and/or short-term survival of new neurons rather than long-term survival or differentiation (Mohapel et al. 2005). Therefore, it might be possible that our experimental paradigm, which allowed newborn cells to mature for 6 weeks, was too long to depict differences between fornix DBS and sham animals. Indeed, in an unpublished data set, we stained brains of fornix DBS and sham animals from a previous behavioral study (Hescham et al. 2013) for doublecortin, a marker of neural precursor cells, and found preliminary evidence for enhanced neurogenesis in the dentate gyrus. Nonetheless, short-term neurogenic differences might not explain the enhanced memory performance 6 weeks after stimulation. We therefore speculate that fornix DBS might have had an effect on other neuroplastic mechanisms, for instance synaptic potentiation or changes in the enzymatic machinery of neurons and terminals, which allow long-lasting functional changes. It has been suggested before that changes in proliferative activity are not necessarily a key factor determining the efficacy of synaptic potentiation (Krugers et al. 2007). In line with this, it was shown that synaptic potentiation was restored within 6 weeks in a transgenic mouse model of suppressed neurogenesis (Singer et al. 2011). Moreover, fornix DBS has shown to upregulate the expression of several neurotrophic factors, such as brain-derived neurotrophic factor (BDNF) and vascular endothelial growth factor (VEGF), but also markers of synaptic plasticity known to play key roles in memory processing, such as nerve growth associated protein 43 (GAP-43), synaptophysin and α-synuclein (Gondard et al. 2015).

In conclusion, our results suggest that acute fornix DBS improves long-term spatial memory independent of DBS-induced neurogenesis, but possibly by increasing the connectivity among existing neurons. Further studies are indicated, which unravel the effects of fornix DBS on long-term potentiation and long-term depression.
